# Mechanochemical Copper-Catalyzed Azide–Alkyne Cycloaddition (CuAAC)

**DOI:** 10.1021/acs.joc.5c02481

**Published:** 2026-01-22

**Authors:** Artur Kasprzak

**Affiliations:** † Faculty of Chemistry, Warsaw University of Technology, Noakowskiego Str. 3, 00-664 Warsaw, Poland

## Abstract

Over the last 15 years, significant progress has been made in developing mechanochemical approaches for the copper-catalyzed azide–alkyne cycloaddition (CuAAC) reaction, utilizing various starting materials and different copper sources and process parameters. This synopsis systematically discusses developments in the mechanochemical CuAAC reaction, aiming not only to highlight the benefits of employing this approach for designing molecules featuring a 1,2,3-triazole motif but also to provide a comprehensive guide for chemists interested in exploring this area and to stimulate future advances in the field.

## Graphical abstract

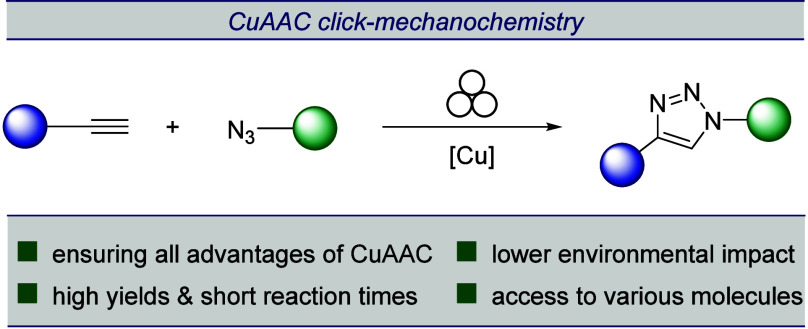

## Introduction

The copper-catalyzed azide–alkyne 1,3-dipolar cycloaddition (CuAAC) utilizing azides and alkynes as starting materials, introduced independently by the groups of Sharpless^1^ and Meldal^2^ in 2002, represents one of the leading click-chemistry methodologies employed in organic chemistry for the synthesis of various functional molecules, starting from pharmaceuticals,^3,4^ up to advanced materials science^5–8^ and site-specific conjugation to biomolecules.^9,10^ From the mechanistic viewpoint, in brief, the terminal alkyne is activated by the copper­(I) salt, which further undergoes cycloaddition with the azide component, forming a 1,4-disubstituted 1,2,3-triazole skeleton.^11,12^ The key features of this approach include high regioselectivity, functional group tolerance, chemoselectivity, compatibility with aqueous or mixed water-organic solvent systems, mild reaction conditions, and high reaction yields.^13,14^

In recent years, mechanochemistry has been demonstrated to be a powerful tool in modern organic synthesis.^15–23^ It offers a sustainable alternative to conventional in-solution methods, together with the perspective of improving reaction yields and time and lowering the negative environmental impact of organic synthesis. It also might provide access to molecules and reaction pathways that are difficult to achieve or cannot be achieved under conventional conditions.^20,24^ Mechanochemical approaches are also scalable and easy to conduct, making them attractive for basic academic research and industrial applications. From the technical viewpoint, typical mechanochemical approaches involve chemical transformations by ball milling, grinding, or mixing using various instruments, such as ball mills, mixing mills, or, rather historically, hand-milling in a mortar.

Over the last 15 years, the reports on click-mechanochemistry reactions have increased constantly, leaving their mark in the development of modern, nonconventional organic synthesis techniques from such perspectives like general metal catalyzed reactions toward small organic molecules,^25,26^ carbohydrate science,^27^ polymer chemistry^28^ or materials science.^29^ This synopsis systematically discusses the reported mechanochemical approaches to the CuAAC reaction (CuAAC click-mechanochemistry), focusing exclusively on their synthetic aspects, highlighting current achievements and potential further developments. Synthetic strategies employed to conduct these processes are summarized and discussed, providing an organized guide for researchers eager to dive into this area of synthetic organic chemistry.

## Discussion

### Preface

Table 1 denotes the graphics used in the figures included in this synopsis to graphically label the type of employed copper source, namely (*i*) copper­(I) (Cu^+^) based (Table 1, entry 1), (*ii*) copper­(II) (Cu^2+^) based (Table 1, entry 2), or (*iii*) copper containing reaction set up component, such as copper powder, vial, balls or nanoparticles (Table 1, entry 3).

**Table 1:**
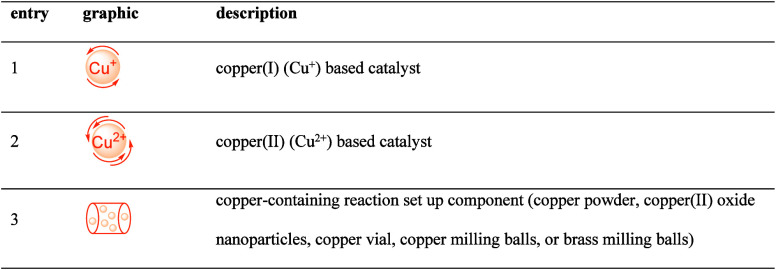
A list of graphics included in all figures in this synopsis used to label the type of copper catalyst employed

The discussion is divided into two sections focusing on CuAAC click-mechanochemistry with (*i*) small organic molecules and (*ii*) organized materials and macromolecules. The achievements within those sections are discussed in chronological order. In addition to the discussion, the timeline of general developments within CuAAC click-mechanochemistry is graphically presented in Chart 1.

**Chart 1:**
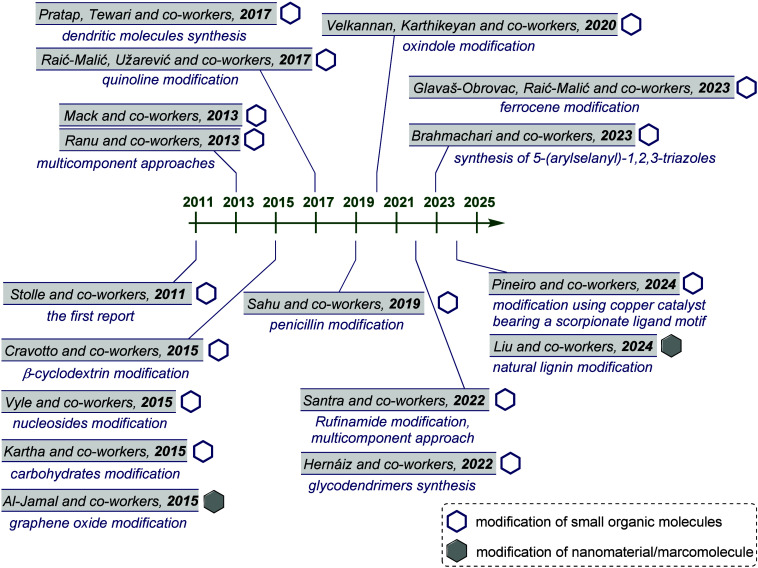
Timeline for Developments of CuAAC Click-Mechanochemistry

Additionally, while no cited paper indicated any severe issues regarding handling potentially explosive azides under mechanochemical conditions, it is highly recommended to take this potential risk into account while conducting any CuAAC click-mechanochemical process. In particular, the need for the implementation of proper personal-protective and occupational safety (*e.g*., laboratory protective screens) strategies should be highlighted. For mechanochemical reactions with sodium azide, it is recommended to perform the process using a grinding jar and balls made from nonreactive materials, such as zirconia, stainless steel, or polytetrafluoroethylene (PTFE), which prevents the formation of reactive metallic species and other reactive impurities during the grinding process. To limit potential heat buildup and localized heating, which should be avoided in processes involving sodium azide, it is also necessary to consider relatively short milling times or intermittent milling procedures, along with careful optimization of the milling frequency and the generated impact force. Finally, it is recommended to use sodium azide in a strict stoichiometric amount and, if possible, perform the one-pot mechanochemical process in the presence of a chemically inert milling auxiliary (*e.g*., Al_2_O_3_) to suppress localized reactivity.

### Mechanochemical Approaches toward CuAAC-Derived Small Organic Molecules

The first report on the mechanochemical approach for the CuAAC reaction was published by Stolle and co-workers in 2011.^30^ The authors performed the reactions in a planetary ball mill (ZrO_2_ milling balls, 6 balls, *d* = 15 mm, and ZrO_2_ 45 mL reaction beaker under solvent-free conditions using copper­(II) acetate (Cu­(OAc)_2_) as a copper source (0.05 equiv) and employing aromatic or aliphatic alkynes and aliphatic azides as starting materials (Figure 1). Fused quartz sand (SiO_2_) was employed as an inert grinding auxiliary. Depending on the transformation, sodium ascorbate was used as a reducing agent (especially in the cases of less active starting materials). The methodology provided high conversion, purity, and regio- and chemoselectivity (Glaser coupling product not observed) in short reaction times (5–20 min). The Authors also demonstrated the potential of the designed methodology for synthesizing new macromolecular architectures, as exemplified by the transformation of propargyl-functionalized polystyrene.

**Figure 1:**
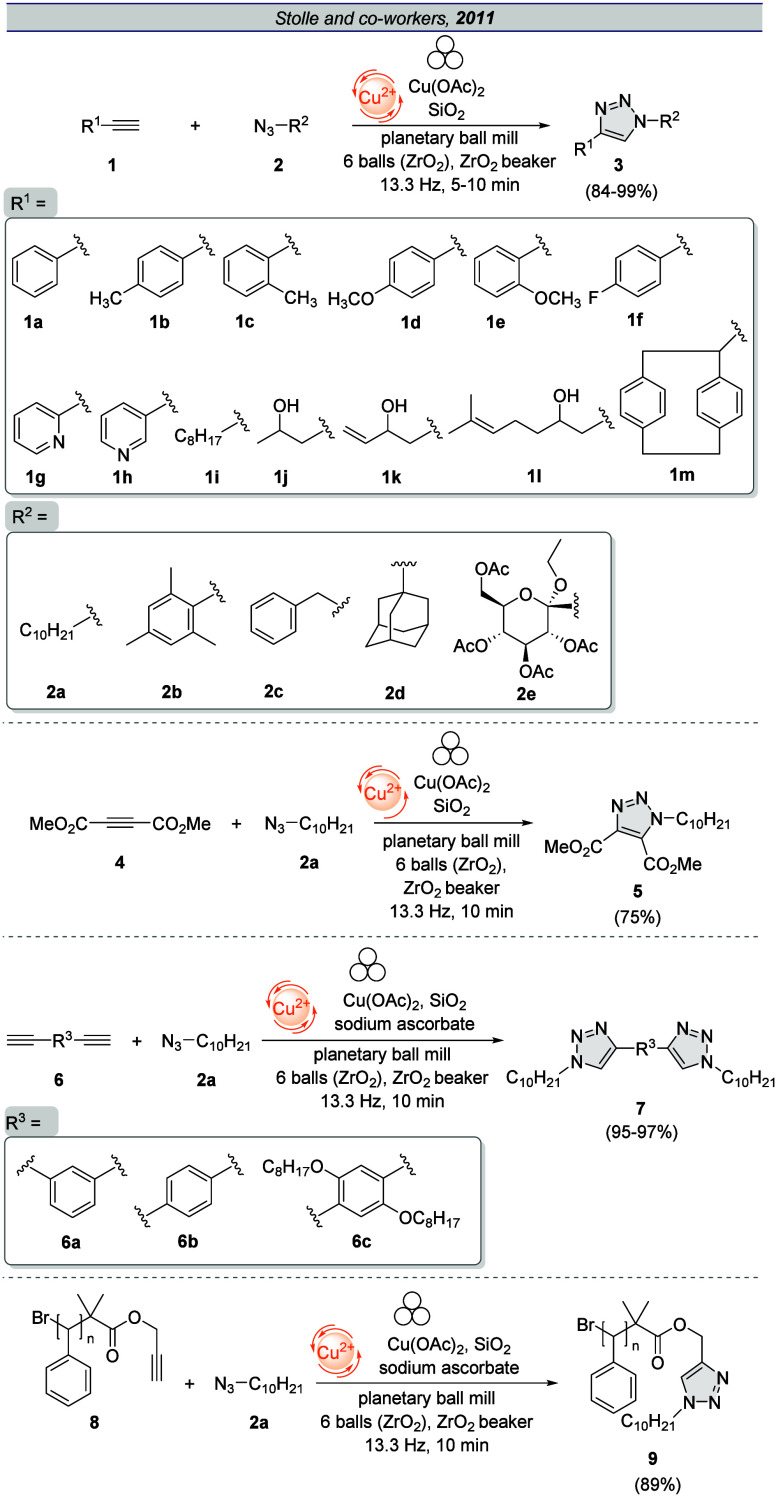
First report on CuAAC click-mechanochemistry.

Regarding further studies on searching fundamental conditions and reaction set-ups for CuAAC click-mechanochemistry, in 2013, two reports on the multicomponent approach toward CuAAC were published independently by the groups of Mack^31^ and Ranu.^32^ In these studies, except the alkyne starting material, benzyl halide derivatives^31,32^ or other aliphatic bromides^32^ were used in line with the sodium azide (NaN_3_) that enabled *in situ* formation of respective azides (Figure 2, Figure 3a). These reports differed in the mechanochemical approach: in the work by Mack and co-workers^31^ 1 copper milling ball (3/16″) in a custom-made copper reaction vessel (2.0″ × 0.5″) was employed, whereas in the report by Ranu and colleagues^32^ previously prepared copper catalyst (0.10 equiv) was used (its preparation was based on the stirring the mixture of copper­(II) sulfate pentahydrate (CuSO_4_·5H_2_O) and alumina oxide (Al_2_O_3_; basic) followed by evaporation and drying; copper content based on ICP-MS analysis was 18.78 mg·g^–1^). In the work by the Ranu group,^32^ the authors also presented an effective (isolated yields of 83–91%) tandem multicomponent approach toward synthesizing 1,2,3-triazoles by CuAAC click-mechanochemistry (6 stainless steel milling balls (*d* = 10 mm) in a 25 mL stainless steel beaker), which employed selected boronic acids as starting materials (Figure 3b), serving as a new concept in this field.

**Figure 2:**
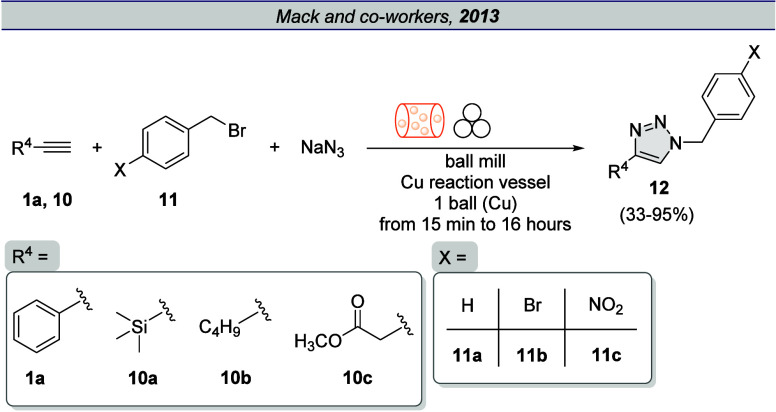
First multicomponent approach toward CuAAC click-mechanochemistry (milling frequency not provided, Spex Certiprep 8000 M mill used for the reaction).

**Figure 3:**
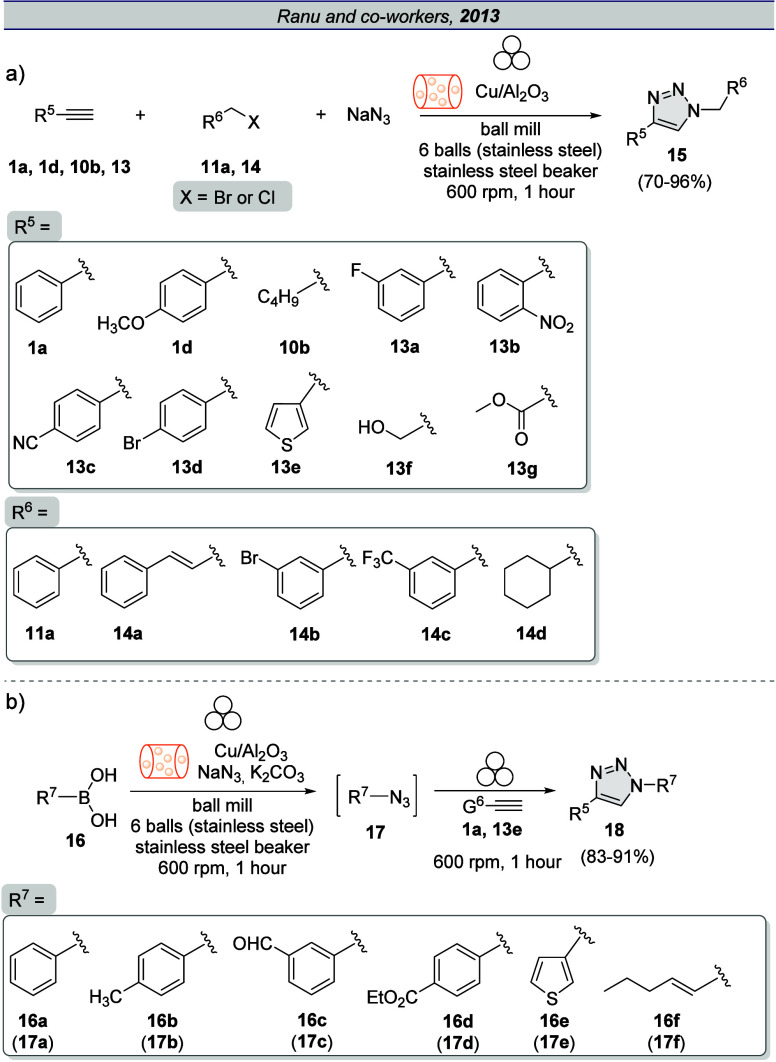
Multicomponent approaches for CuAAC click-mechanochemistry starting from (**a**) halides, (**b**) boronic acids.

Another variation for CuAAC click-mechanochemistry conditions was reported in 2015 by the group of Cravotto.^33^ In this approach, the Authors used copper powder (1 equiv) as a copper source, and the milling process was conducted using stainless steel milling balls (1500 balls, *d* = 2 mm and 48 balls, *d* = 5 mm) in a 50 mL stainless steel beaker (Figure 4a). This methodology provided target 1,2,3-triazoles in high yields (88–99%) in only 5–10 min. Notably, the wider utility of this process (even on a gram scale) was demonstrated by the successful CuAAC click-mechanochemical reaction (30 min of milling with 0.1 equiv of Cu powder) starting from β-cyclodextrin monoazide (**22**; Figure 4b).

**Figure 4:**
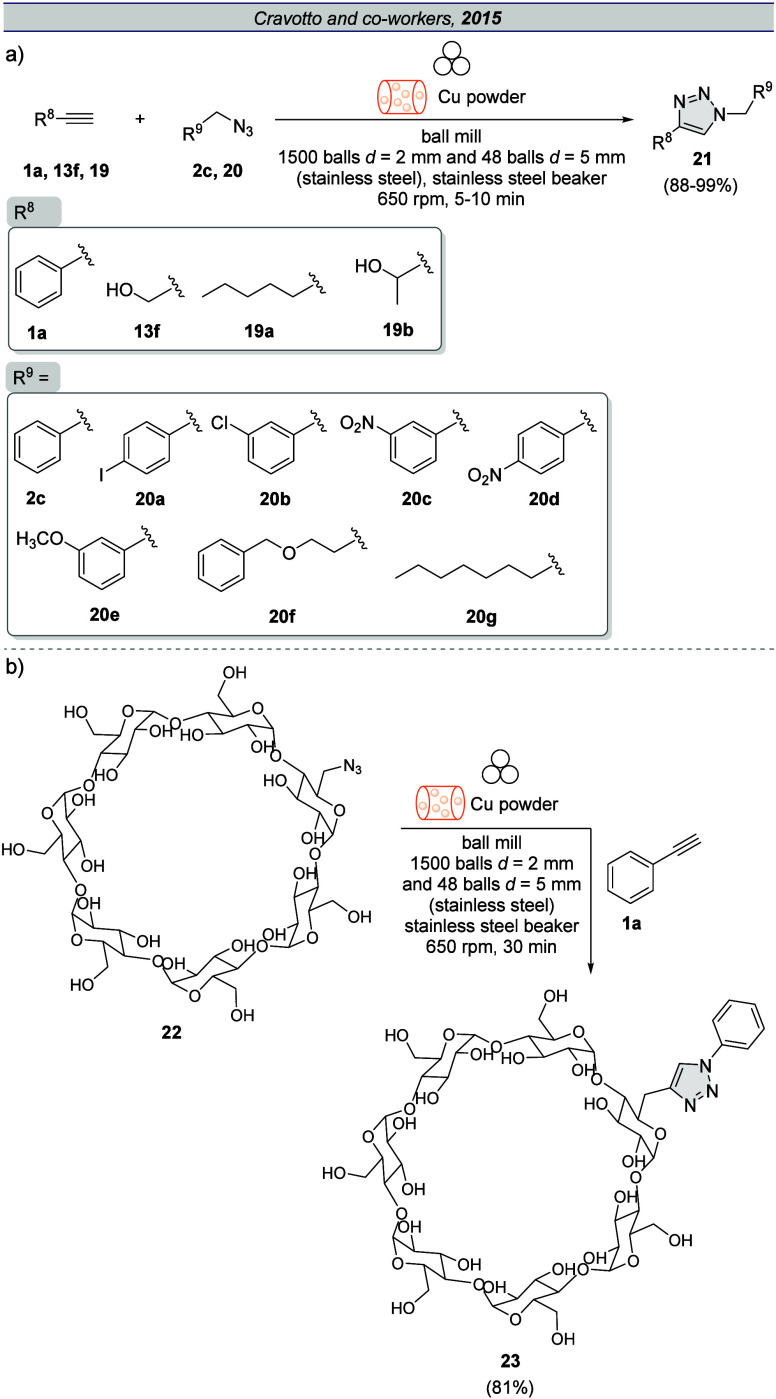
CuAAC click-mechanochemistry reaction with (**a**) benzyl azide derivatives or aliphatic azides, (**b**) β-cyclodextrin monoazide.

In 2015, a mechanochemical liquid-assisted grinding (LAG) with ethyl acetate was introduced by Vyle and co-workers^34^ to the modification of nucleosides. 5′-Azido-5′-deoxythymidine (**5′-AZT**, Figure 5) and mono-*N*-propargylamide substituted azobenzenes (**24**) were used as starting materials. A mechanochemical process in a high-speed vibration ball mill (HSVBM) filled with 1 copper milling ball (3/32″) in a custom-made copper vial (2.0″ × 0.5″, internal volume 5 mL) yielded the desired 1,2,3-triazoles (**25**; 63–80%). It is worth noting that HSVBM and the conventional (vibratory) ball mill operate on related principles, but HSVBM features higher vibration frequencies together with commonly smaller amplitudes than the ball mill, which might result in more intense force impact and higher energy input per time unit. Notably, contrary to the classical in-solution methods, the mechanochemical CuAAC approach did not result in a copper ion contamination of products (when the process was employed under low energy impact conditions), providing an attractive synthetic alternative in the chemistry of such biologically relevant molecules.

**Figure 5:**
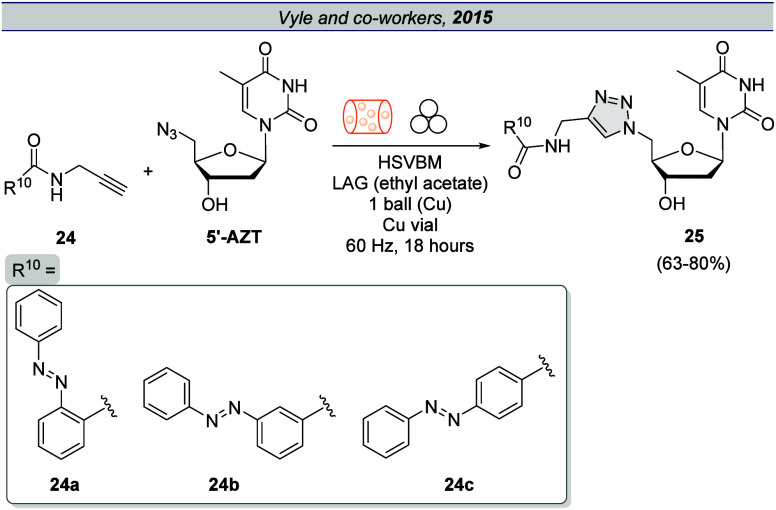
Mechanochemical CuAAC reactions starting from 5′-azido-5′-deoxythymidine **24**.

In the same year (2015), the group of Kartha^35,36^ reported on the two works on the CuAAC click-mechanochemical approach that utilized carbohydrate derivatives as starting materials. In the first report,^35^ the authors demonstrated that by performing the reaction in a planetary ball mill (10 stainless steel milling balls, *d* = 10 mm in a 50 mL stainless steel jar) and employing CuSO_4_·5H_2_O in line with sodium ascorbate, it is possible to transform propargyl derivatives of carbohydrates (**26**; Figure 6a) into the corresponding 1,2,3-triazole-containing lipids (**28**). The second work^36^ focused on synthesizing molecules comprising several functionalized sugar moieties (**29–33**; Figure 6b), showing wider applicability of the developed method. The Authors also investigated the self-assembly properties of the resultant molecules (more detailed discussion could be considered beyond the scope of this synopsis).

**Figure 6:**
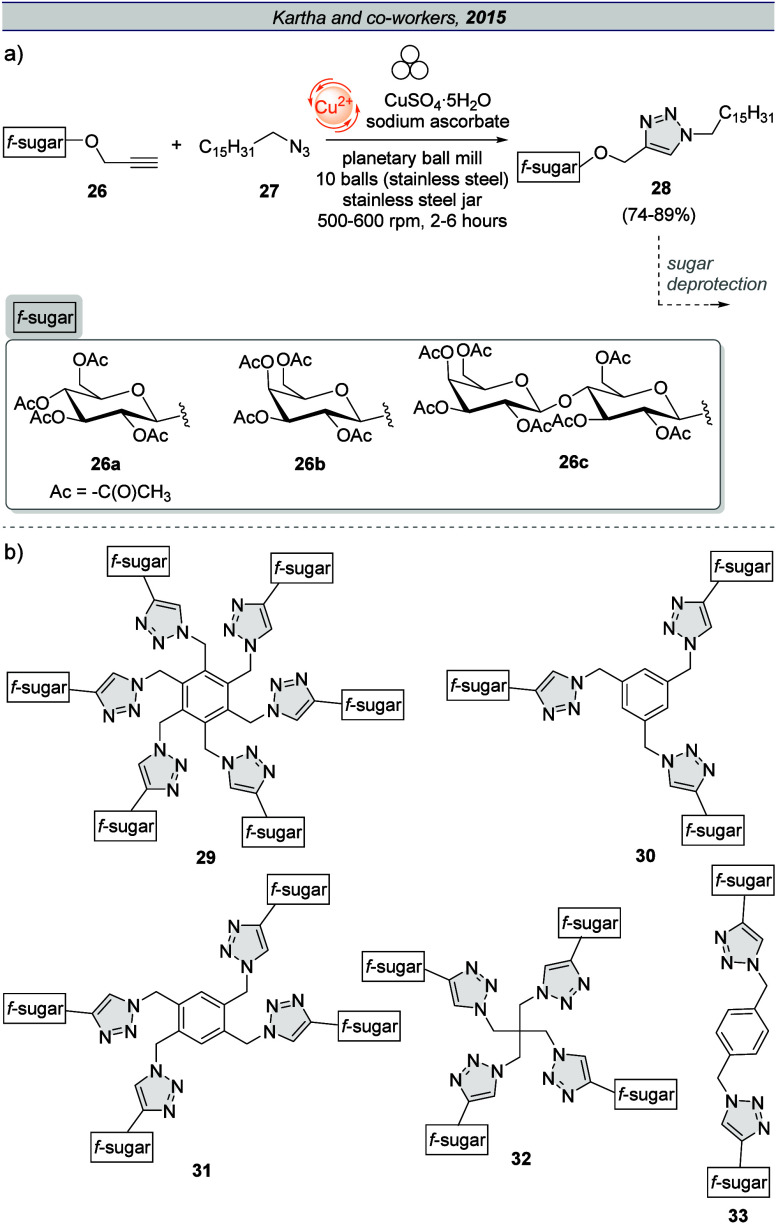
CuAAC click-mechanochemistry derived synthesis of a 1,2,3-triazole containing sugar moiety: (**a**) derivatization of carbohydrates, (**b**) graphical representation of the molecules comprising several sugar moieties.

In 2017, Raić-Malić, Užarević and co-workers^37^ reported on the CuAAC click-mechanochemical synthesis of quinoline derivatives. Propargylated quinoline derivative **34** (Figure 7; prepared by the classical in-solution method) and phenyl azide or its halogenated derivatives (**17a**, **35**) were used as starting materials. Three ball milling strategies (polytetrafluoroethylene (PTFE) vessel) were tested, namely, employing the following as a copper source: (*i*) copper­(II) acetate monohydrate (Cu­(OAc)_2_·H_2_O; 2 stainless steel milling balls *d* = 7 mm), (*ii*) copper­(I) iodide (CuI) in the presence of *N*,*N*-diisopropylethylamine (DIPEA; 2 stainless steel milling balls *d* = 7 mm), or (*iii*) 2 brass milling balls (*d* = 7 mm). The second approach provided the highest reaction yields (Figure 7). Interestingly, regarding the halide substituent in the 4-position of phenyl azide, the reactivity trend was as follows: H (**17a**; 79% yield) < Cl (**35a**; 85% yield) < Br (**35b**; 87% yield) < I (**35c**; 92% yield), demonstrating some effects of the electronegativity of the halide on the reaction outcomes.

**Figure 7:**
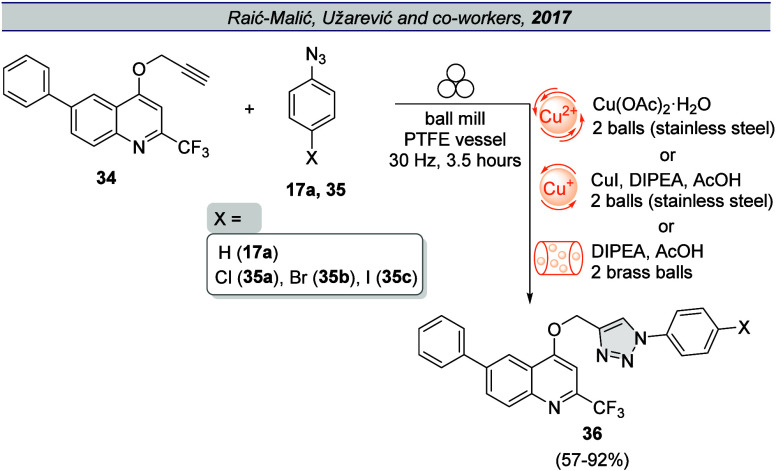
Mechanochemical CuAAC reaction with quinoline derivative.

Sulfonated, dendritic-like molecules comprising 1,2,3-triazole skeletons were synthesized in 2017 by the team of Pratap and Tewari.^38^ For the mechanochemical reaction (LAG approach with water), the authors have used the newly designed, nonhygroscopic and air-stable copper catalyst **39** (Figure 8). 2,4,6-Tris­(prop-2-yn-1-ylthio)-1,3,5-triazine **37** and the respective sulfonated *N*,*N*-substituted derivatives of 2-azidoethan-1-amine **38** were used as starting materials, proving access to several dendrimers **40**. Compared to the in-solution methods, the mechanochemical process (grinding) provided higher conversion of starting materials and improved reaction yields (94–99%) in shorter reaction times.

**Figure 8:**
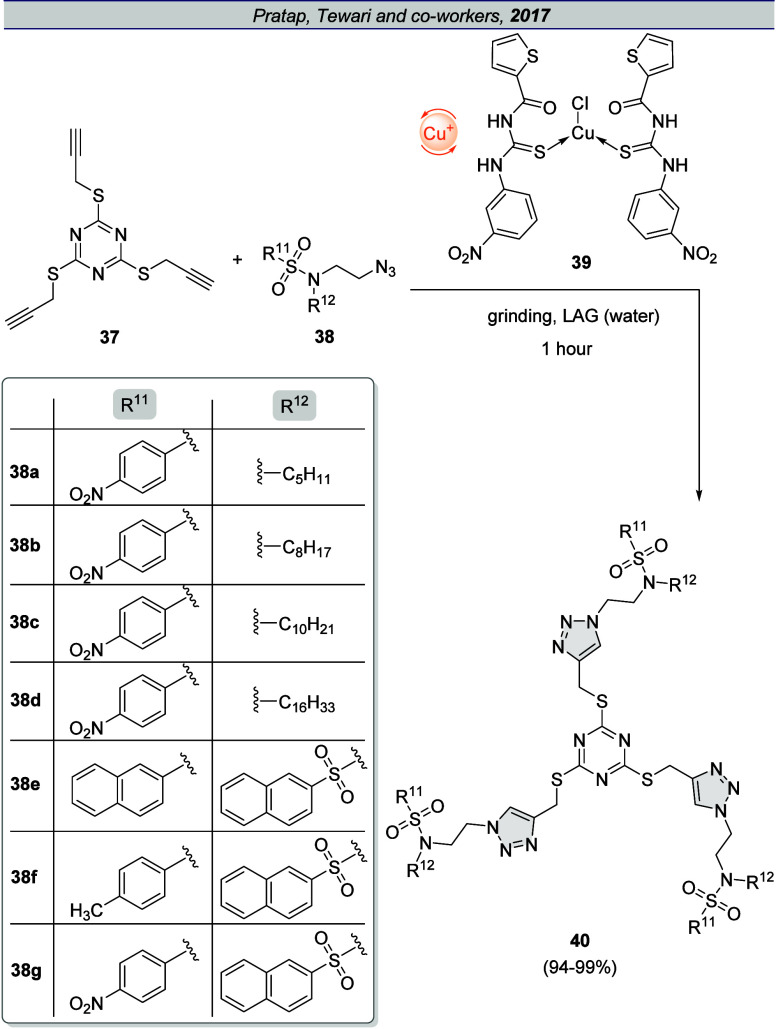
Mechanochemical CuAAC approach toward dendritic-like molecules (grinding instrument not provided).

CuAAC click-mechanochemical modification of antibiotics, taking β-lactam antibiotic penicillin as a representative case, was introduced by Sahu and co-workers^39^ in 2019. Azide-containing penicillin derivative **42** (Figure 9; synthesized by the classical in-solution method) and aromatic and aliphatic alkynes (**1d**, **41**) were selected as starting materials. Respective 1,2,3-triazoles **43** were obtained in isolated yields between 57% and 85% in 3 h. Grinding in a planetary ball mill (10 stainless steel milling balls, *d* = 10 mm in a 50 mL stainless steel jar) was employed with copper­(II) sulfate (CuSO_4_; 0.2 equiv) as a copper source in the presence of sodium ascorbate. From an application perspective, attractive antibacterial properties of the resultant molecules were concluded. Overall, this report, for the first time, reported CuAAC click-mechanochemical modification of a small organic molecule with well-established biological action, demonstrating the wider applicability of this approach and potential relevance to the pharmaceutical industry.

**Figure 9:**
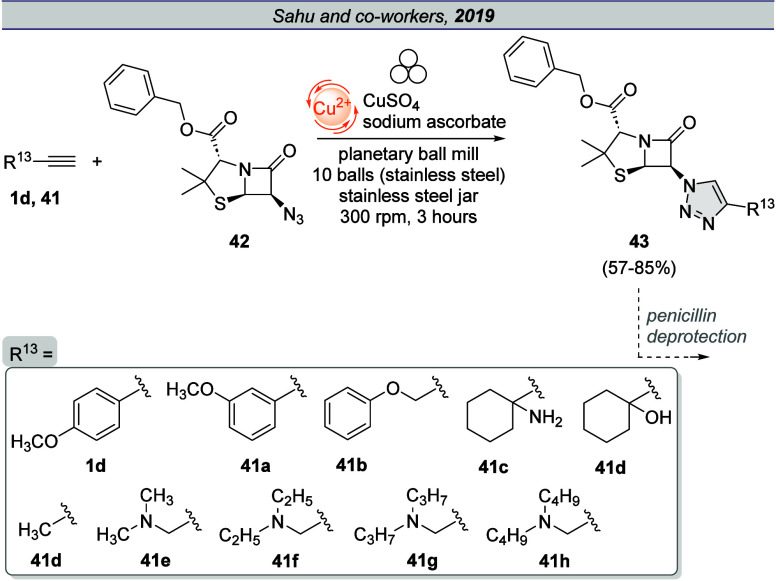
Derivatization of a penicillin derivative using the CuAAC click-mechanochemical approach.

The mechanochemical synthesis employing the CuAAC approach of other biologically active molecules (antimicrobial agents) was reported in 2020 by Velkannan, Karthikeyan and co-workers.^40^ These molecules featured the presence of an oxindole scaffold and were synthesized in a multicomponent reaction, which employed 1-(prop-2-yn-1-yl)­indoline-2,3-dione **44** (Figure 10) and benzyl azide or its derivatives (**2c, 20d, 45**) as starting materials. A ball milling process (6 ZrO_2_ milling balls (6 × 15 mm) and ZrO_2_, 45 mL reaction beaker) was conducted with the usage of copper­(II) oxide nanoparticles (CuO NPs; 0.05 equiv) in the presence of 1,4-diazabicyclo[2.2.2]­octane (DABCO). The process provided very high isolated yields of the resultant oxindoles **47** (87–92%) in a short reaction time (30 min).

**Figure 10:**
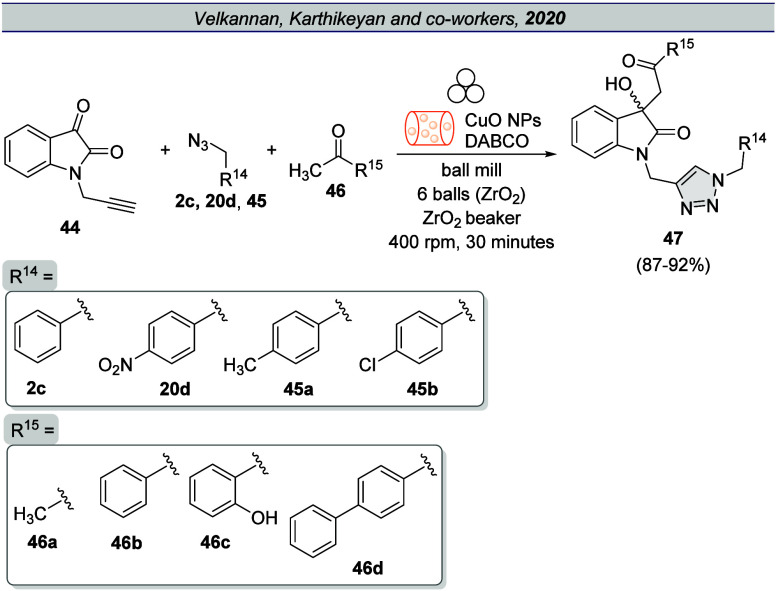
CuAAC click-mechanochemical approach toward oxindole derivatization.

The work reported in 2022 by the group of Santra^41^ on the synthesis of Rufinamide (antiseizure drug; **56b**, Figure 11) could be considered as a continuation of interest in applying CuAAC click-mechanochemistry for the modification of biologically active molecules. The experimental conditions for this process were based on the screening experiments that revealed that the treatment of aromatic or heterocyclic alkynes (**1a-b, 13e, 48**; Figure 11) with the derivatives bearing an α-bromocarbonyl motif (**49**; Figure 11a; in the presence of sodium azide) or *p*-toluenesulfonyl azide **51** (Figure 11b) provided target 1,4-substituted 1,2,3-triazoles (**50**, **52**, **55**) in high isolated yields (42–96%). Five copper beads (as milling balls; 0.27/0.27′′) were used as a copper source, and the process was performed under HSVBM conditions (1.5–3.5 h of milling; 25 mL stainless steel milling bowl). The optimized conditions were applied to synthesize Rufinamide ethyl ester derivative **55b** (76% yield for Rufinamide, after the reaction with ammonium hydroxide; Figure 11c) or its chloro-analog **55a**. This work demonstrated that the CuAAC click-mechanochemical approach could serve as an interesting alternative for synthesizing active pharmaceutical ingredients (APIs).

**Figure 11:**
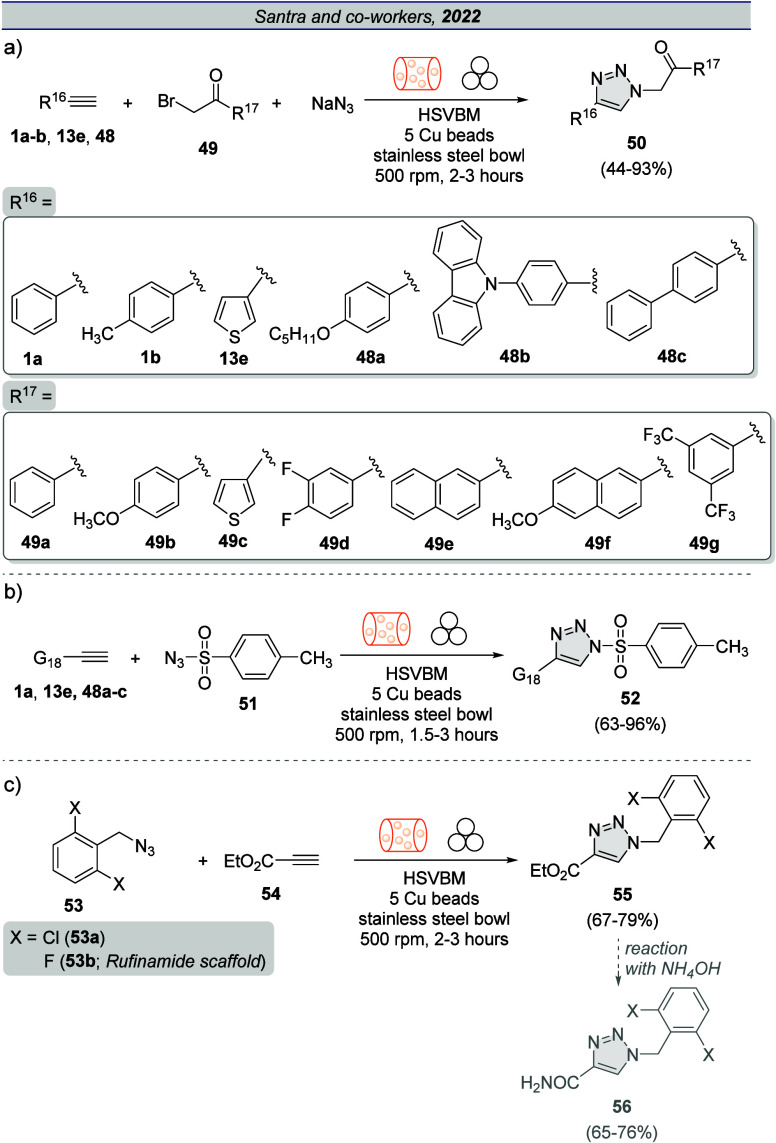
CuAAC click-mechanochemical reaction (**a**) utilizing derivatives bearing an α-bromocarbonyl motif, (**b**) starting from *p*-toluenesulfonyl azide, (**c**) synthesis of Rufinamide and its chloro-analog.

In 2022, the group of Hernáiz^42^ reported on the CuAAC click-mechanochemical approach toward synthesizing dendrimers bearing glucuronic acid residues. The azide-derivative of glucuronic acid methyl ester (**57**; Figure 12) and the respective propargylated resorcin derivatives substituted at the 5 position (**58–59**) were used as starting materials. A ball milling process (planetary ball mill; 200 balls, *d* = 3 mm or 30 balls *d* = 5 mm (stainless steel), 12 mL stainless steel jar) with CuSO_4_·5H_2_O as a copper source, and in the presence of sodium ascorbate and SiO_2_ as grinding auxiliary, provided almost full conversions (>99%) in 11 h (isolated yield: 65–70%). The mechanochemical reaction outcomes were more satisfactory than the classical in-solution synthesis (conversion <66%). The authors also compared the effectiveness of the respective reactions conducted under microwave (MW) conditions. While the same conversion was observed in a shorter reaction time (90 min) together with a slightly higher isolated yield (80%), this approach required the use of organic solvent for the process (*N*,*N*-dimethylacetamide; DMA), in contrast to the solvent-free CuAAC click-mechanochemical approach.

**Figure 12:**
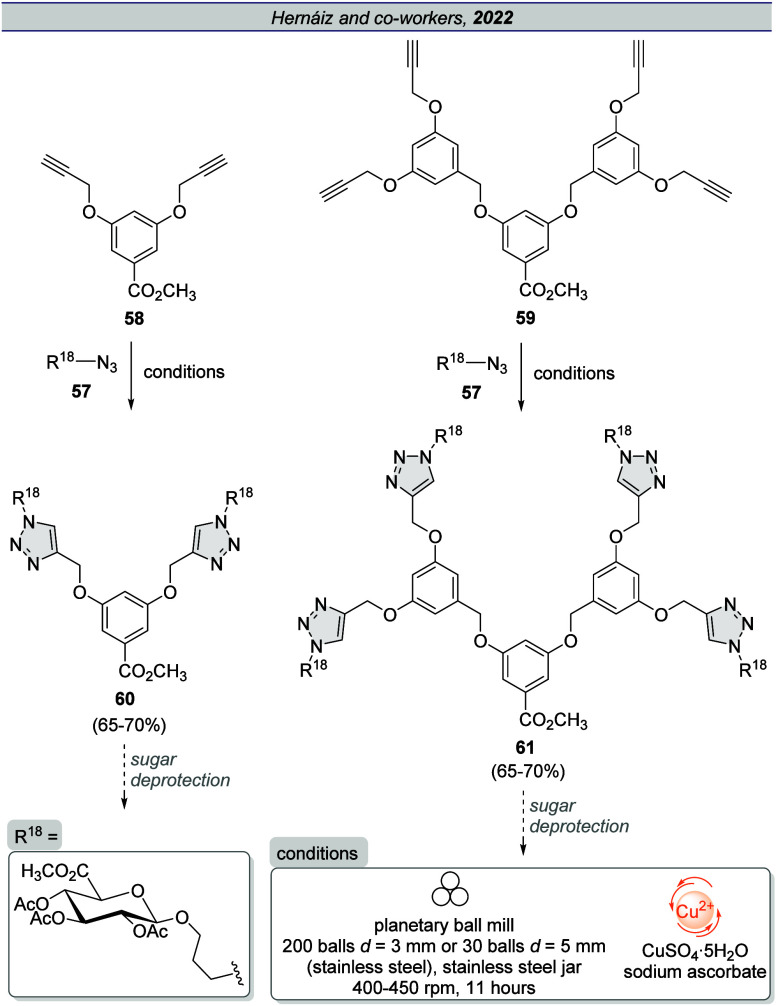
CuAAC click-mechanochemical synthesis of glucuronic acid glycodendrimers.

A mechanochemical synthesis of a series of 5-(arylselanyl)-1,2,3-triazoles (**65**; Figure 13) was reported in 2023 by Brahmachari and co-workers.^43^ The designed multicomponent reaction employed the following as starting materials: (*i*) various phenylacetylene derivatives (**1a**–**b**, **1d**, **1f**, **13d**, **48c**, **62**) or 2-ethynylthiophene (**13e**), (*ii*) benzyl bromide derivatives (**2c**, **63**), (*iii*) diaryl diselenides (**64**), and (*iv*) sodium azide. The HSVBM process (6 balls (stainless steel) *d* = 10 mm, 25 mL stainless steel jar) in the presence of CuI (0.1 equiv) as a copper source (1,10-phenanotroline and basic alumina also added) provided high isolated yields (42–87%) of target molecules in a short reaction time (15 min). Along with the broad scope of starting materials (see Figure 13), the authors concluded improved green chemistry parameters of the designed approach compared to the classical in-solution approach, expressed by such parameters as high carbon efficiency, low waste generation, and operational simplicity.

**Figure 13:**
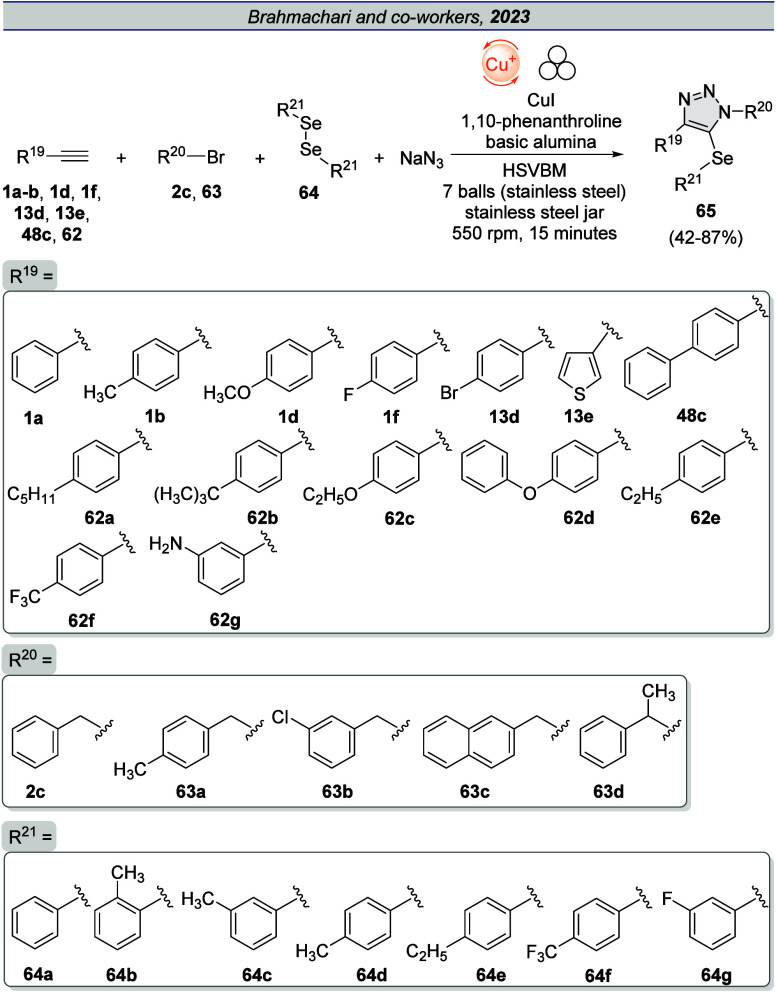
5-(Arylselanyl)-1,2,3-triazoles synthesized under CuAAC click-mechanochemical conditions.

The first organometallic derivatives synthesized using the CuAAC click-mechanochemical approach, namely the conjugates of ferrocene with quinoline or quinolone (**69–70**; Figure 14), were reported in 2023 by the group of Glavaš-Obrovac, Raić-Malić and co-workers.^44^ 1,1′-Disubstituted ferrocene azides (**66**) and propargylated quinoline/quinolone derivatives (**67–68**) were used as the starting materials. As compared to the classical in-solution method (same copper source used), mechanochemistry with Cu­(OAc)_2_ (0.05 equiv) as a copper source (LAG approach using methanol; 2 balls (stainless steel), *d* = 7 mm, stainless steel vessel) provided higher reaction yields of target derivatives (improvement between 10 and 50%) in shorter reaction times (reduction from 24 h to 30 min). The resultant ferrocene derivatives were also studied regarding their antiproliferative properties (a detailed discussion on this aspect of work could be considered beyond the scope of this synopsis).

**Figure 14:**
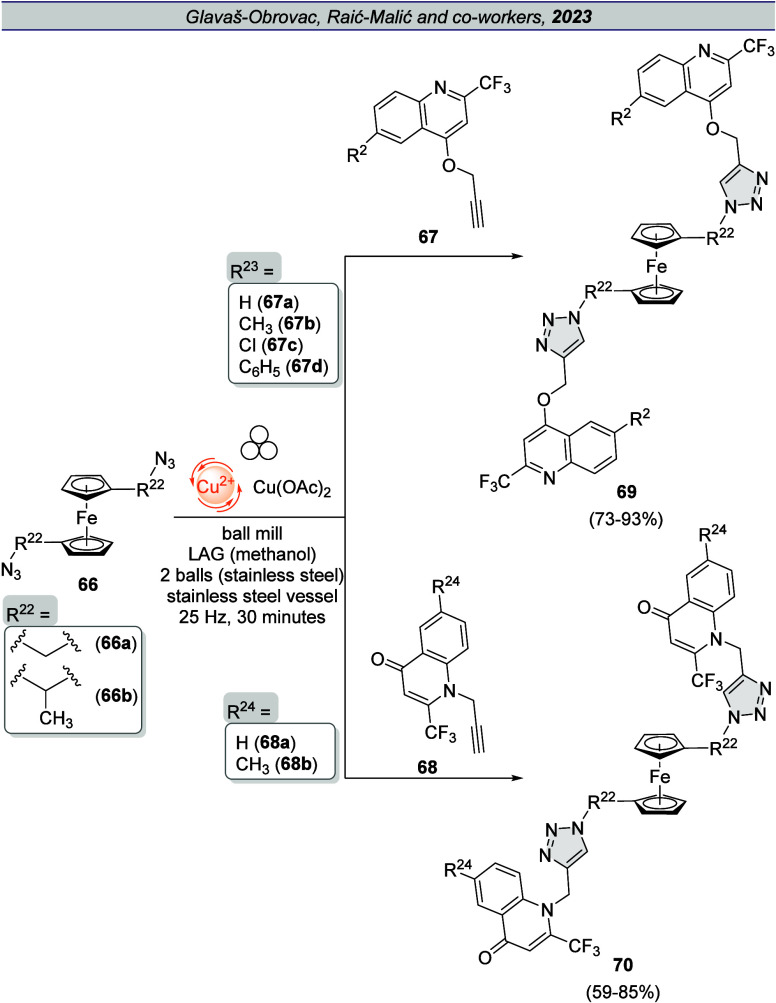
Conjugates of ferrocene with quinoline/quinolone synthesized under CuAAC click-mechanochemical conditions.

In 2024 the group of Pineiro^45^ reported on studies on the usage of a copper­(II) catalyst bearing a scorpionate ligand motif (**72**; Figure 15) for the effective CuAAC click-mechanochemical synthesis of selected 1,2,3-triazoles (**73**) starting from benzyl bromide (**11a**) or aromatic or aliphatic acetylene derivatives (**1a-b, 10a, 19b, 71**) in the presence of sodium azide. Notably, the ligand motif of the designed catalyst was also synthesized mechanochemically (ball milling; 2 balls (stainless steel), *d* = 7 mm, 10 mL stainless steel jar). The designed catalyst (0.015 equiv) improved the synthesis yield in comparison to previously reported methods employing other classical or nonclassical reaction conditions and various copper catalysts, along with greener reaction parameters, as demonstrated by the sustainability metrics analyses, such as atom economy,^46^ *E*-Factor^47^ or EcoScale^48^ factors. This study laid the foundation for future research on designing new copper catalysts for CuAAC click-mechanochemistry reactions for basic and more sophisticated CuAAC reactions.

**Figure 15:**
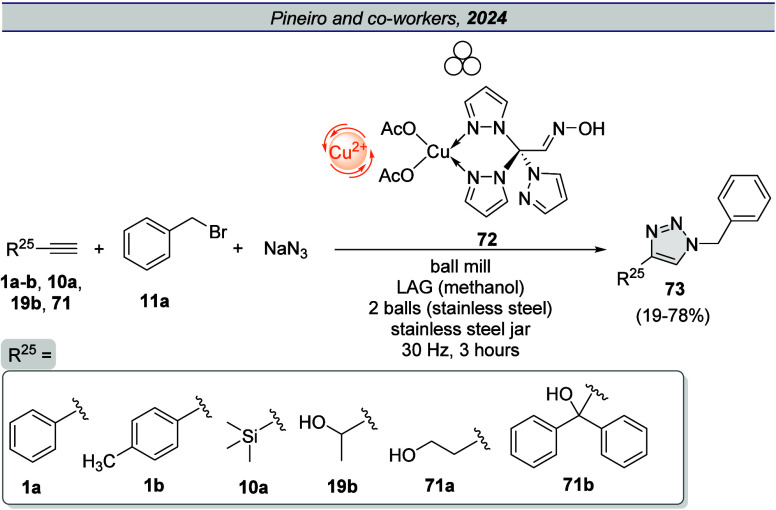
Synthesis of 1,2,3-triazoles using the CuAAC click-mechanochemical approach with a copper catalyst bearing a scorpionate ligand motif. The catalyst that provided the most satisfactory reaction outcomes is presented; other catalysts can be found in the source publication.

### Mechanochemical Approaches to CuAAC-Derived Organized Materials and Macromolecules

The CuAAC click-mechanochemistry approach could also be implemented in the nonconventional modification of functional materials and macromolecules. Two works focused on such a modification approach have been published.

In 2015 the group of Al-Jamal^49^ demonstrated that relatively simple CuAAC click-mechanochemistry conditions (CuSO_4_, sodium ascorbate) could be applied for the modification of a graphene oxide (GO) derivative bearing propargyl groups (**74**; Figure 16, a partial structure of GO in Figure 16 was presented according to the literature^50,51^ ). Poly­(ethylene glycol) (PEG) derivative bearing maleimide and azide end-groups (**75**) was used as an azide component in the reaction. The milling strategy (mixer mill, 1 h; 4 balls (stainless steel), *d* = 10 mm, 10 mL stainless steel jar) provided access to a 1,2,3-triazole containing GO derivative (**76**) which, thanks to the presence of a reactive maleimide group, was further bioconjugated to the antibody for breast cancer cells targeting purposes. This work is an example of how reaction CuAAC click-mechanochemistry conditions for small organic molecules could be implemented in materials science.

**Figure 16:**
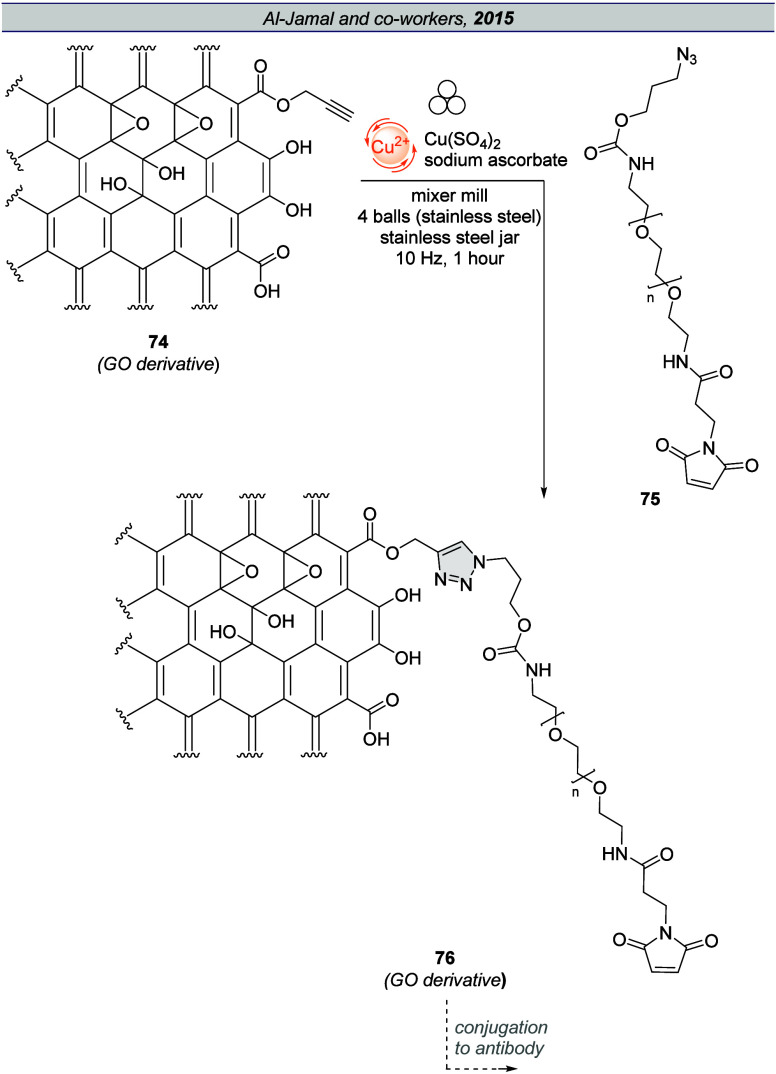
CuAAC click-mechanochemical approach for the GO derivatization.

In 2024, the group of Liu^52^ reported on the CuAAC click-mechanochemical modification of lignin. The mechanochemical reaction (planetary ball mill, 30 min; 40 balls (ZrO_2_), *d* = 3 mm, 45 mL ZrO_2_ milling jar) was based on the Cu­(OAc)_2_ (0.08 equiv) catalyzed derivatization of propargylated natural lignin (compound **77**; Figure 17) with various azides (**2c**, **78**). The reaction conditions were optimized based on the test experiments with ethyl 2-azidoacetate (**78a**) and lignin’s model compounds (phenolic compounds). Conversion for each lignin modification ranged between 45 and 92.5%, whereas the yield was 37–77% (provided in two cases). Notably, the designed lignin modification protocol not only enabled avoiding the use of solvents but also prevented lignin from degradation. This report demonstrated the wider applicability of CuAAC click-mechanochemistry for modifying macromolecules.

**Figure 17:**
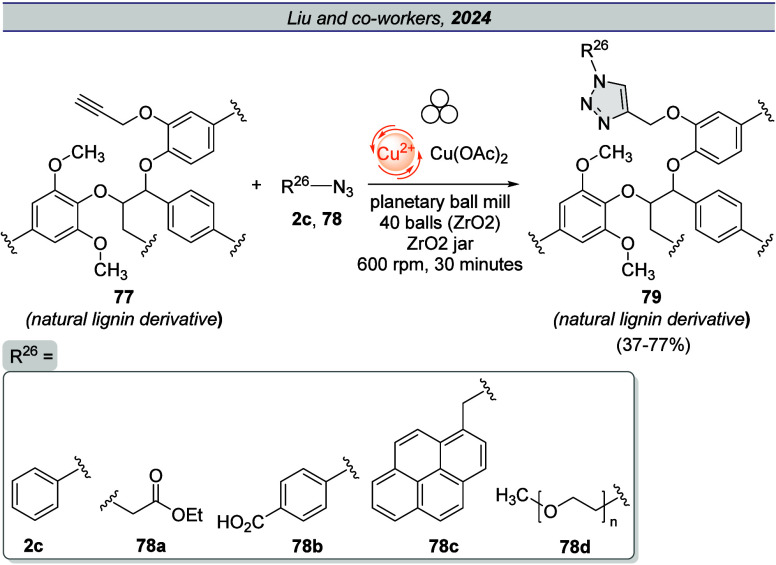
Natural lignin modification using the CuAAC click-mechanochemical approach. The partial structure of natural lignin is presented according to the source paper.

## Summary and Conclusions

The copper-catalyzed azide–alkyne 1,3-dipolar cycloaddition (CuAAC) click-mechanochemical approaches have developed from synthesizing relatively simple organic compounds, through the derivatization of various classes of small organic molecules (like carbohydrates and nucleosides) up to materials science (modification of graphene oxide and lignin). To the best of the authors’ knowledge, this synopsis describes all reported protocols for the CuAAC reaction performed under mechanochemical conditions, bringing together the published studies into a single systematic overview from the perspective of organic chemistry. The known examples include various starting materials and different copper sources and process parameters. To organize this wide range of examples, Table 2 summarizes the reported approaches. This tabular summary might serve as a guide to researchers considering process parameters for running CuAAC click-mechanochemistry reactions as a greener alternative to the classical in-solution approach. The general guidelines in terms of designing a CuAAC click-mechanochemistry process, beyond changing common grinding parameters, such as grinding time, ball material/size, milling frequency or testing LAG approach, may include (*i*) screening appropriate copper sources, with initial trials recommended using commonly employed Cu^+^ or Cu^2+^ salts, (*ii*) testing the addition of a chemically inert grinding auxiliary, particularly when at least one of the reaction components is liquid or viscous, and (*iii*) changing the starting material type, as *in situ* generation of an azide component from the respective organic halide has been reported to provide good reaction outcomes.

**Table 2:** Summary of Key Information for the Developed Approaches of CuAAC Click-Mechanochemical Reactions

Figure in this synopsis	Starting material type - alkyne	The second starting material(s) type	Copper source	Molar equiv of copper source	Instrument to conduct a mechanochemical process	Reaction time	Yield (%)	Ref
Figure 1	various benzene derivatives, various aliphatic derivatives	various aliphatic and aromatic azides	Cu(OAc)_2_ ^a^	0.05	planetary ball mill (ZrO_2_ beaker and milling balls)	5–20 min	75–97^b^	^30^
Figure 2	various benzene derivatives, various aliphatic derivatives	benzyl bromide derivatives, sodium azide	copper reaction vessel with copper milling balls	N/A	ball mill	16 h	33–95^b^	^31^
Figure 3a	various benzene derivatives, various aliphatic derivatives	benzyl halide derivatives, secondary halides, sodium azide	Cu/Al_2_O_3_ ^c^	0.01	ball mill	16 h	70–96^d^	^32^
Figure 3b	various benzene derivatives, various aliphatic derivatives	various boronic acid derivatives, sodium azide	Cu/Al_2_O_3_ ^c^ ^,^ ^e^	0.01	ball mill	2 h	83–91^d^	^32^
Figure 4a	ethynylbenzene, various aliphatic derivatives	octyl azide, various aliphatic azides	Cu powder	1.00	planetary ball mill	5–10 min	88–99^d^	^33^
Figure 4b	ethynylbenzene	β-cyclodextrin monoazide	Cu powder	0.1	planetary ball mill	30 min	81^d^	^33^
Figure 5	mono-*N*-propargylamide substituted azobenzenes	5′-azido-5**′**-deoxythymidine	copper milling balls	N/A	HSVBM^f^ (LAG, ethyl acetate)	18 h	63–80^d^ ^,^ ^g^	^34^
Figure 6a	propargylated sugars	hexadecyl azide	CuSO_4_·5H_2_O^h^	0.2	planetary ball mill	2–6 h	74–89^d^	^35^
Figure 6b	propargylated sugars	various aliphatic azides	CuSO_4_·5H_2_O^h^	0.2	planetary ball mill	N/A	85–92^d^	^36^
Figure 7	propargylated quinoline derivatives	phenyl azide, p-halogenated benzyl azide	Cu(OAc)_2_·H_2_O	0.05	ball mill	3.5 h	57–77^d^	^37^
			CuI^i^	0.02			79–92^d^	
			brass balls^i^	N/A			76–97^d^	
Figure 8	dendritic-like triazine derivative	sulfonated, aliphatic azides	presynthesized copper catalyst (see structure in Figure 8)	0.06	not specified (LAG, water)	1 h	94–99^d^	^38^
Figure 9	various benzene derivatives, various aliphatic derivatives	azide derivative of penicillin	CuSO_4_ ^h^	0.2	planetary ball mill	3 h	57–85^d^	^39^
Figure 10	propargyl-containing oxindole derivatives	benzyl azide or its derivatives	CuO nanoparticles^j^	0.05	ball mill^k^	30 min	87–92^d^	^40^
Figure 11a	aromatic or heterocyclic alkynes	derivatives bearing an α-bromo­carbonyl motif	copper beads	N/A	HSVBM^f^	2–3 h	44–93^d^	^41^
Figure 11b	aromatic or heterocyclic alkynes	*p*-toluene­sulfonyl azide	copper beads	N/A	HSVBM^f^	1.5–3 h	63–83^d^	^41^
Figure 11c	ethyl propiolate	derivatives of Rufinamide bearing an azide group	copper beads	N/A	HSVBM^f^	2 h	67–79^d^	^41^
Figure 12	respective propargylated resorcine derivatives (dendritic)	azide-derivative of glucuronic acid methyl ester	CuSO_4_·5H_2_O^h^	0.2	planetary ball mill	11 h	65–70^d^	^42^
Figure 13	various phenyl­acetylene derivatives, 2-ethynyl­thiophene	benzyl bromide derivatives, sodium azide, diaryl diselenides^l^	CuI^m^	0.1	HSVBM^f^	15 min	42–87^d^	^43^
Figure 14	propargylated quinoline/quinolone derivatives	1,1′-disubstituted ferrocene azides	Cu(OAc)_2_	0.05	ball mill (LAG, methanol)	30 min	59–93^d^	^44^
Figure 15	aromatic or aliphatic alkynes	benzyl bromide, sodium azide	presynthesized copper catalyst (see structure in Figure 15)	0.015	ball mill	3 h	19–78^d^	^45^
Figure 16	propargylated GO^n^	PEG derivative bearing maleimide and azide end-groups	CuSO_4_ ^h^	0.06 (with respect to the azide)	mixer mill	1 h	-^o^	^49^
Figure 17	natural lignin propargyl derivative^p^	aliphatic or aromatic azides	Cu(OAc)_2_	0.08	planetary ball mill (ZrO_2_ milling balls)	30 min	37–77^q^	^50^

a Inert milling auxiliary = SiO_2_ (quartz sand), sodium ascorbate used in selected experiments.

b Estimated by^1^H NMR.

c Prepared by stirring the mixture of CuSO_4_·5H_2_O with basic alumina, followed by evaporation and drying.

d Isolated yields.

e Potassium carbonate (K_2_CO_3_) added in the first reaction step.

f High speed vibration mill (HSVBM).

g No copper ions contamination of products in comparison to in-solution method.

h Sodium ascorbate added.

i DIPEA, AcOH added.

j DABCO added.

k ZrO_2_ milling balls.

l One-pot reaction.

m 1,10-Phenanotroline and basic alumina also added.

n Graphene oxide (GO).

o GO derivative obtained; thus, no yield is provided.

p Lignin model compounds (phenolic compounds, see details in the source paper) were also subjected to the process.

q Isolated yields provided for two reactions, conversions for all reactions between 45 and 92.5%.

Considering its broad substrate scope and various commercially available or easy-to-prepare copper catalysts, CuAAC click-mechanochemistry represents an attractive strategy potentially applicable to a wide range of novel organic processes, including multistep reactions. The increasing number of reports might be anticipated in the coming years within the CuAAC click-mechanochemistry of small organic molecules and applied materials science, where organic chemistry plays a key role in materials’ synthesis. In particular, in terms of small organic molecule chemistry, CuAAC click-mechanochemistry might offer attractive perspectives in terms of small-scale or industrial synthesis of functional organic molecules, such as active pharmaceutical ingredients (APIs), where lowering the generated organic waste in the process together with providing improved reaction performance are remarkably important. Second, taking into account the still growing importance of designing nanomaterials for various applications, CuAAC click-mechanochemistry holds a significant potential in revolutionizing approaches for nanomaterial synthesis and/or modification. The major challenges in this concept might include evaluating the scope of this approach in terms of its plausible effect, if any, on the morphology or other relevant properties of the material under mechanochemical conditions. In addition, CuAAC click-mechanochemistry related research could also be an interesting concept within other areas of chemistry, as was demonstrated by the studies on the mechanochemical retro-cycloaddition of 1,2,3-triazoles,^53,54^ mechanochemical activation of the metal–organic framework for CuAAC purposes,^55,56^ or stress sensing system applications^57,58^ (interested readers proficient in Chinese could also investigate the following paper^59^).

To conclude, the field of CuAAC click-mechanochemistry holds significant potential for advancing modern organic chemistry, providing a robust synthetic foundation for further important achievements in the field of chemistry.

## Data Availability

The data underlying this study are available in the published article.
